# Clival Osteomyelitis in an Adult Patient: A Case Report

**DOI:** 10.7759/cureus.65055

**Published:** 2024-07-21

**Authors:** Vinod Shinde, Aishwarya Kothari, Mayur Ingale, Sunanda Devi Putta

**Affiliations:** 1 Otorhinolaryngology, Dr. D. Y. Patil Medical College, Hospital and Research Centre, Dr. D. Y. Patil Vidyapeeth (Deemed to be University) Pune, Pune, IND

**Keywords:** paranasal sinus infection, atlanto-axial joint, mri, clival abscess, skull-base osteomyelitis

## Abstract

Clival osteomyelitis is an uncommon skull base infection that mostly affects elderly diabetics and is frequently caused by malignant otitis externa or paranasal sinus infections. It manifests as severe otalgia, fever, auditory fullness, and purulent otorrhea. Clinical history, physical examination, test data, radiographic findings, and pathogen identification all contribute to a diagnosis. Treatment consists of extended intravenous broad-spectrum antibiotics, with severe cases necessitating surgical debridement. We present a case of a 20-year-old girl with bilateral ear discharge, nasal blockage, and purulent rhinorrhea, as well as a dull neck ache increased by extension. An MRI revealed osteomyelitis in the clivus and right atlanto-occipital joint. The clival abscess was drained transnasally using endoscopic techniques. Microbiological tests revealed Streptococcus intermedius. The post-operative recovery was uneventful, with extended antibiotic therapy. Early identification and treatment are critical for preventing serious consequences, as illustrated in this case, where surgical and antibiotic care improves patient outcomes.

## Introduction

Osteomyelitis may infect any bones in the body, although long bones are more typically affected [1]. Clival osteomyelitis is an uncommon skull base infection that is typically caused by malignant otitis externa and, in a few instances, paranasal sinus infection [2]. Older diabetic individuals with malignant otitis externa are more likely to develop osteomyelitis at the base of their skull [3].

In skull base osteomyelitis (SBO), severe and deep ear pain affects the side of the head, upper side of the head, behind the ear, and around the eye, along with fever, a sensation of ear fullness, and foul, purulent ear discharge [4]. SBO can be diagnosed on the basis of the patient's medical history, clinical assessment, lab findings, imaging studies, and microbiological identification at the infection site [5].

The main treatment for SBO is culture-directed, prolonged intravenous broad-spectrum antibiotics. Extensive soft-tissue involvement requires aggressive surgical removal of infected tissue [6].

## Case presentation

A 20-year-old female presented with symptoms of bilateral ear discharge: yellowish in color, purulent in consistency, moderate in amount, non-foul-smelling, and non-blood-tinged. It was gradual in onset and progressive in nature over the course of two years. There was a history of bilateral nasal obstruction and purulent rhinorrhea from the right nasal cavity, which was intermittent, insidious in onset, and gradually progressive over a period of 15 days. The patient also gave a history of pain over the nape of the neck, which was continuous and dull. The pain aggravated during the extension of the neck and was temporarily relieved by taking medication. There was no history of a long-standing chronic illness or blood transfusion. Past and family history were not contributory.

**Figure 1:**
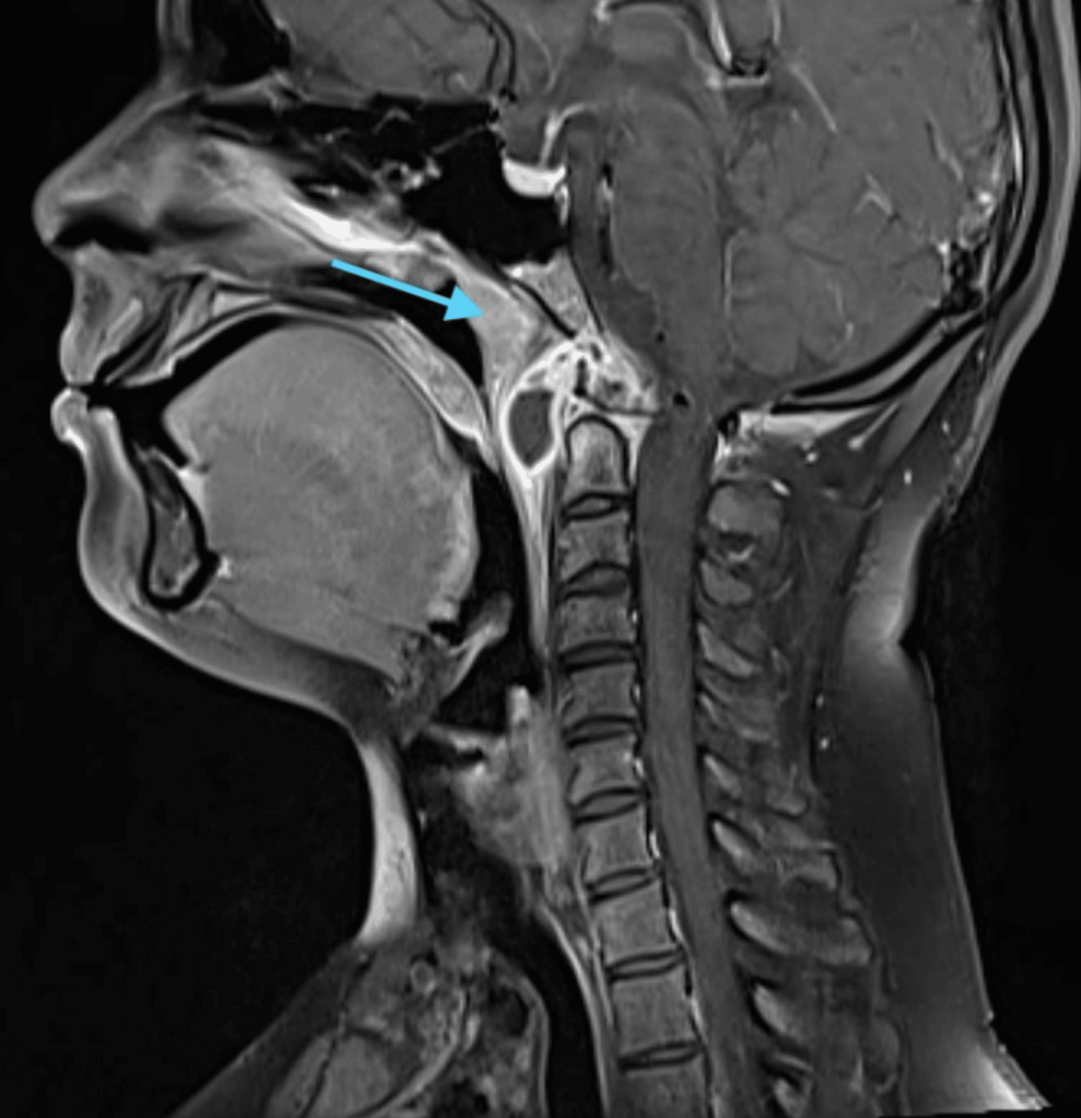
MRI cranio-vertebral junction - sagittal plane Altered marrow signals are noted in the clivus, anterior arch of the C1 vertebra, its right lateral mass, adjoining posterior neural arch, dens, and adjoining body of the C2 vertebra, which appear hypointense on T1WI. Erosion and destruction are noted involving the clivus (blue arrow).

**Figure 2:**
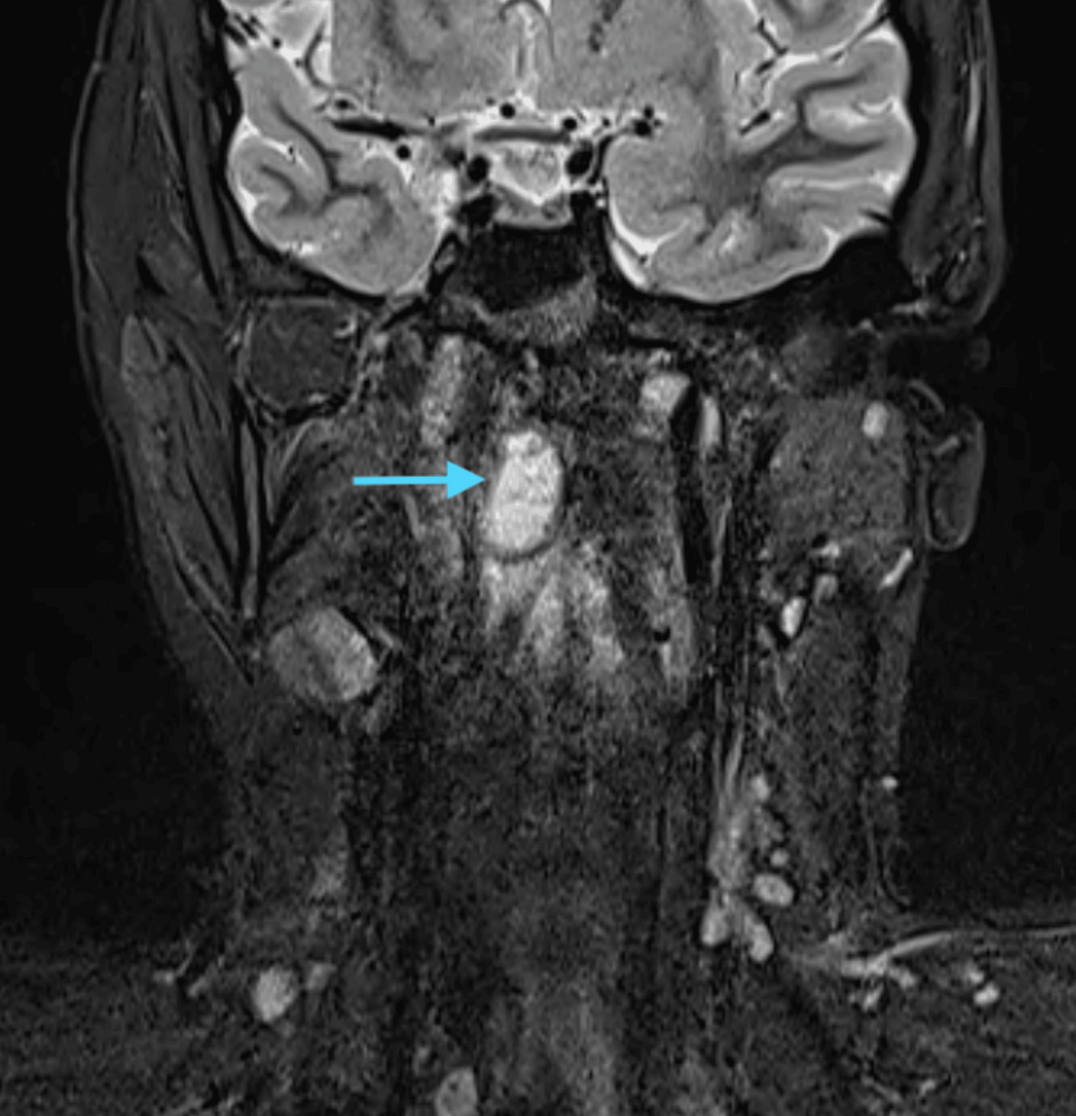
MRI cranio-vertebral junction (contrast) - coronal plane A well-defined lesion measuring approximately 12 x 13 x 26 mm (AP x TR x CC) (blue arrow) is noted in prevertebral soft tissues just on the right side of the midline at the C1-C2 level. It appears hyperintense on STIR and shows peripheral rim enhancement with central non-enhancing hypointense areas suggestive of liquefaction.

**Figure 3:**
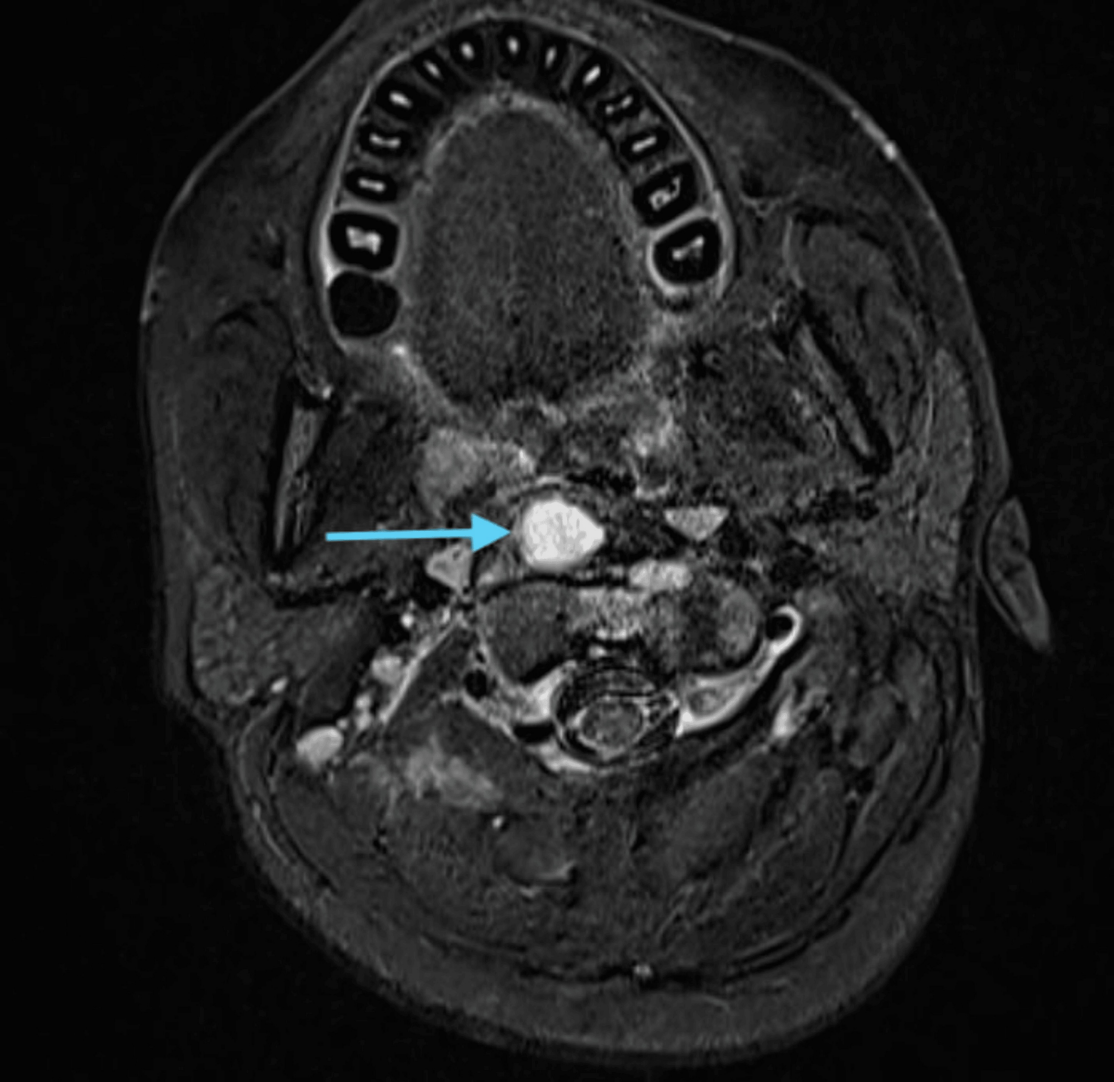
MRI cranio-vertebral junction (contrast) - axial plane A heterogeneously soft tissue component measuring approximately 20 x 36 x 2 mm (AP x TR x CC) (blue arrow) is noted in relation to the foramen magnum on the right side and the right atlanto-occipital joint, right lateral atlanto-axial joint with osteomyelitis affecting clivus, and central non-enhancing hypointense areas of liquefaction or necrosis.

All routine blood investigations were done, and the hemogram was suggestive of lymphocytosis with a raised erythrocyte sedimentation rate (ESR). A diagnostic nasal endoscopy was done, which was suggestive of nasal mucosal congestion bilaterally with a deviated nasal septum towards the left side, and there was nasopharyngeal purulent discharge from the right nasal cavity. The MRI cranio-vertebral junction was suggestive of an infective etiology involving the right atlanto-occipital joint and the right lateral atlanto-axial joint, with osteomyelitis affecting the clivus, as seen in the sagittal plane (Figure 1), coronal plane with contrast (Figure 2), and and axial plane with contrast (Figure 3).

After obtaining fitness for surgery under general anesthesia by the anesthesiologist and written informed consent from the patient, under the coverage of broad-spectrum antibiotics, the patient was taken up for incision and drainage of the clival abscess via a trans-nasal endoscopic surgical approach.

The abscess was drained using a stab incision. Further, the aspirate from the abscess was sent for microbiological testing. Hemostasis was achieved, and the post-operative period was uneventful. Microbiological testing revealed the presence of Gram-positive cocci in the chain, suggestive of Streptococcus intermedius. The tuberculin test was negative. The patient was started on antibiotics according to the culture reports, and a prolonged course of antibiotic treatment for 14 days was given. The patient was symptomatically better with no clinical signs of infection.

## Discussion

The term "osteomyelitis" was introduced by Nelaton in 1844 [1]. Osteomyelitis is defined as an inflammatory bone condition produced mostly by pyogenic organisms that can affect one or more elements of a bone, such as the cortex, periosteum, bone marrow, and surrounding soft tissues [7]. Infection within the clivus bone is a kind of skull-base osteomyelitis [2].

The illness is more prevalent in the white population (69.3%) compared to Native Americans (13.2%), African Americans (6.5%), and Asians (2.9%) [8]. Clivus osteomyelitis symptoms are often vague. Some of the more prevalent symptoms are headaches and cranial nerve palsies. Furthermore, patients often have immunocompromised conditions, such as diabetes [9].

Despite substantial investigation, the specific pathophysiology of SBO is still unknown. The majority of instances occur in elderly diabetics and immunocompromised persons who have diseases that cause an alkaline pH in the cerumen, reduced immunological responses, hypoperfusion, and microangiopathy, all of which predispose them to SBO. Water irrigation for cerumen removal increases susceptibility to Pseudomonas infection in elderly diabetics by lowering ear wax acidity and lysozyme levels. Malignant otitis externa can migrate from the external auditory canal (EAC) to the skull base via Santorini fissures and the osseocartilaginous junction, potentially causing cranial neuropathies, parotid gland involvement, and temporomandibular joint dysfunction. Inferomedial spread may impact the carotid artery and jugular bulb, whereas posterior spread may include the sigmoid sinus. Untreated infections can result in numerous abscesses, pneumatized trabeculated bone, and cervical spine involvement [10].

Pseudomonas aeruginosa is the most prevalent infection associated with osteomyelitis, caused by malignant otitis externa. This appears to be true for SBO, while additional species have been identified, including aspergillus, Gram-positive organisms, mycobacterium, and candida [7].

Blood testing can be used to help diagnose SBO. Acute-phase reactants, notably the ESR, will be regularly high. Additionally, biopsy material can be sent for microbiological examination [11]. Early CT scans of skull base osteomyelitis show soft tissue inflammation and fat plane effacement in the subtemporal triangle. Advanced illness will result in abscess development and aggressive bone deterioration. While magnetic resonance imaging is more effective than CT for detecting soft tissue changes, it is not useful for monitoring disease progression [12].

The treatment of SBO begins with long-term, broad-spectrum antibiotic medicines. However, it is then critical to collect culture results from the problematic organism and switch agents to target it appropriately. Piperacillin-tazobactam, ceftazidime, and ciprofloxacin are examples of commonly used antibacterial therapies, depending on the infecting bacterium. Some antifungal regimens utilized are the lipid amphotericin B formulation, voriconazole, posaconazole, voriconazole, itraconazole, and caspofungin [2]. Surgical therapy is also advised if an abscess has developed in the clivus bone's joint space or if there is severe bone damage [13].

SBO has been associated with considerable disability and fatality [2]. SBO can lead to consequences such as thrombosis of the lateral sinus and internal jugular vein, meningitis, eye muscle paralysis, blindness, cervical spine erosion, and cranial nerve paralysis [3].

Early identification and treatment are important for saving the lives of SBO patients. Rapid action, such as surgical intervention and antibiotic therapy, resulted in improved results for our patients.

## Conclusions

This case emphasizes the necessity of identifying clival osteomyelitis in individuals who have prolonged ear drainage and neck discomfort. Early detection by imaging and timely management with surgical drainage and tailored antibiotic medication are critical for optimal results. The discovery of Streptococcus intermedius in this case emphasizes the importance of microbiological testing to guide therapy. Rapid and adequate care can prevent serious consequences, stressing the need for early medical and surgical intervention in clival osteomyelitis.
